# Albuminuria without Manifest Organic Renal Lesions

**Published:** 1901-07

**Authors:** W. A. Deas

**Affiliations:** Richmond, Va.


### ALBUMINURIA WITHOUT MANIFEST ORGANIC RENAL LESIONS.*

BY W. A. DEAS, M.D.,

RICHMOND, VA.

The subject of my paper to-night is "Albuminuria without Manifest Organic Renal Lesions," or Functional Albuminuria, as it is generally called.

Perhaps I ought to offer an apology for presenting a subject which has been so frequently discussed; and yet when we remember in what a very undecided state this subject of transient or functional albuminuria still remains, and how much has been written upon it, and how very little determined, I do not think an apology is necessary for again introducing it. It seems to me that it is a

*Read before the Richmond Academy of Medicine and Surgery, March 12, 1901.

subject ever new and ever interesting to the general practitioner, the specialist, and particularly so to the examiners and medical directors for life insurance companies.

The medical profession has long been familiar with that class of patients in which this form of albuminuria appears. They are apparently in good health, but still they pass either constantly, or from time to time, small, or even large, amounts of albumin in their urine; and this may extend over periods of months or even years. These albuminurias have received many names by different writers. They have been called functional, paroxysmal, intermittent, cyclic, chronic and even physiological.

Our first clear idea of the clinical value of albumin in the urine is due to Dr. Bright of England, in his masterly "Report of Medical Cases" in 1827 and 1831. For a long time following these reports, when albumin or any proteid body was found in the urine, such cases were considered as due to a true renal affection or nephritis, and the gravest prognosis was given. At the present time, although there has been some reaction from those extreme views, and although we know in an examination of urine we may find several other proteid bodies albuminous, still we are all, I think, more or less inclined to look upon any case of albuminuria with suspicion and uneasiness. Following the writings of Dr. Bright, Sir Robert Christison was the first to point out that certain articles of diet caused temporary albuminuria. Following him in 1864, Iaccoud asserted that persistent albuminuria might and did occur in persons in otherwise good health. Since then many other physicians in Europe and America have confirmed the above observations.

Before going further let me say a few words as to the anatomy and physiology of those parts of the kidney which are believed to be concerned in the production of albumin; namely the vessels, the blood and the secreting apparatus. The kidneys are well supplied with arterial blood by the renal arteries. Branches of these arteries give off a large arterial twig to each Malpighian body. Each twig of the afferent artery pierces the capsule, and breaks up into a plexus, the Malpighian tuft or glomerulus; and it is believed by many observers that the glomeruli are directly concerned in the secretion of albumin. From this tuft arises the venous radicles of the efferent veins, which are much smaller than the afferent artery. This of course would tend to renal congestion. The efferent vein, after leaving the capsule of Bowman, breaks up into a plexus, and loosely surrounds the nearest convoluted tubules, leaving spaces generally filled with fluid between the walls of the vessels and the urinary tubules. From this plexus arise the branches forming the venous trunks. The renal veins have no valves, and this is another reason tending to renal congestion. We know that the urinary tubules begin as Bowman's capsule, with its constricted neck; then soon dilate and become convoluted, then pass downward as the descending limb of Henle's loop, then upward again as the ascending limb, becoming convoluted again, and forming irregular tubules; and finally enter the straight or collecting tubes which carry their contents to the pelvis. From the experiments of Haidenbraim, Overbeck and others, which can only be referred to here, it is believed that albumin is a secretion of the epithelial cells of the glomeruli, and takes place within the capsule of Bowman; and in order to secrete the albumin there must be a slowing down of the blood while passing through the tufts.

What we mean then by "Albuminuria without Manifest Organic Lesions," or functional albuminuria, is an albuminuria extending over a longer or shorter period of time, without detection of kidney trouble, and accompanied, in most instances, by fair, or even robust, general health; and with final disappearance of albumin. Many competent observers have made extended tests as to the frequency of these cases. Their results are not at all uniform. They range from two to eighty-four per cent. of the cases examined. This is a great difference, and it is difficult to account for it. It has been ascribed to the varying conditions of seasons, climate, habits, pursuits and surroundings, and to a greater or less degree of care and accuracy in the examinations. Senator and a few others claimed that their examinations led them to believe that albumin could be found in the urine of every healthy person. Sir Wm. Roberts has said that concentrated urine, from persons in undoubted health, were rarely free from traces of albumin. If this be so, it shows how near we all are to a true albuminuria. But I do not think that we are as yet prepared to accept the existence of a true, physiological albuminuria.

These albuminurias have had numerous groupings. Perhaps one of the simplest is this: 1. Simple albuminuria. 2. Uric acid or oxaluric form. 3. Neurotic form.

Before speaking of these forms it might be asked, "Can we say that there is a true physiological albuminuria?" That is, is albumin a constant element in healthy urines. I have just mentioned the conflicting views as to the frequency of functional albuminuria, and shall only now remind you that some observers and many of the insurance companies found in the very many thousand cases examined but two per cent. Others claimed 84 per cent.; and Senator insisted that albumin exists at all times and in all urines, as could be proved by sufficiently delicate tests. Admitting that the insurance companies and others finding only two per cent. may not have used the most delicate tests, still that would not have accounted for the absence of albumin in ninety-eight per cent. of the cases examined. In the cases of those who found eighty-four per cent. of albumin, and of Senator, who found albumin in all urines, it has been claimed by Millard that their tests, as published, were faulty, and it seems to me that we have as yet no sufficient proof that albumin does exist as a normal ingredient in healthy urines.

Another question that might be asked is this: "Are these albuminurias produced in any other way than by some derangement of the circulation, or glandular apparatus of the kidneys?" I will refer to this question again.

And now a few words upon the first form, or simple albuminuria. This form includes those cases which may continue for years in those in apparently good health, and who may never have had any disturbance of digestion, or circulation, or in their nervous functions. They might, perhaps, never have been discovered, except accidentally by the family physician or by an examiner for life insurance. These are considered true functional or chronic albuminurias; and are frequently found to exist after a simple meal, or after eating certain articles of diet; also after exposure to cold or cold bathing; after great muscular, or even very moderate exercise, and after mental strain or brain-work. A record of experiments is a rather dull subject to most hearers, and I will try not to weary you. We are all familiar with those on both animals and man, in which eggs and other albuminous food were given freely by the mouth with the result of producing albuminuria. Grainger Stewart's experiments show that a diet largely of eggs has usually produced albumin in the urine, but in small amount, not egg-albumin. We cannot account for the appearance of this serum-albumin by supposing that the egg-albumin had been changed by digestion into serum-albumin, and then secreted by the kidneys. Semmola experimented on dogs by introducing egg-albumin by the stomach, veins and subcutaneously. The animals were killed, and he always found a true glomerulo-nephritis. It is believed that in all these cases of albuminuria, after a diet of nitrogenous food, including gluten, casein and albumin, some disorder of digestion was produced; and that peptonization was weakened, and that the albuminoid was absorbed in some changed condition, and circulated in the blood as a toxin, and by its irritating qualities excited a glomerulo-nephritis. These cases were dietetic albuminurias, and evidently associated with vascular changes in the kidney; and it has not as yet been demonstrated that such an albuminuria can occur independently of such changes. So far then as our knowledge goes, it is thought to be quite probable that albuminurias not due to glandular changes in the kidneys can be explained by the conditions just mentioned, viz.: irritating albuminoids or toxins in the blood; causing vascular changes in the kidneys. I have already mentioned muscular exercise as causing these albuminurias.

Great stress has been placed upon this cause. The simplest form seems to be what is called postural albuminuria. In these cases when quiet, or lying down, albumin was not found. When up, and after taking even the gentlest exercise, albumin appeared. When lying down again the albumin again disappeared. Dr. Raikes, physician to the Rugby School, England, has reported striking cases. It appears that two mornings in the week, at 7 A.M., the boys were required to attend prayers in the hall, where they had to stand for fifteen minutes. Upon other days they were required to attend prayers in the chapel, where they were seated. It was quite common for the boys to faint in the hall, but not in the chapel. The doctor found that the urine of the boys who were affected always contained albumin, and that they had hard rigid pulses. He thought the trouble was due to a pure hyperemia of the kidneys. The hearts in these cases, he thought, might have been somewhat weakened by fasting since the day before, and this may have caused cerebral amenia, and some vasomotor influence causing hyperemia in the kidneys. When seats were provided in the hall at prayers all trouble ceased. Pavy gives an account of a young English student who stood a civil service examination, and was rejected because albumin was found in his urine. It seemed to be a functional case, and he was afterwards passed. He then went up to Oxford, and after that had to go up for a final physical examination before going to India. He had read up on these cases, and remained in bed until just before the examination. No albumin was found, and he was accepted, there being a temporary disappearance of the albumin. This is another case of postural albuminuria, in which the erect posture seemed to act as violent exercise in others. The cause here has been attributed to altered blood-pressure in the Malpighian tufts. We need not dwell on the albuminuria found in that large class of cases including football players, bicycle riders, athletes, as well as those undergoing less violent exercise of prolonged walking, riding, running and marching. In the cases of twenty-nine football players examined by Dr. Macfarlane, he found albumin in all, from a trace, to a ring a quarter of an inch deep; and in nearly all there were casts, either hyalin, granular or epithelial; and in a few blood cast. Oxalates and urates and uric acid were also there in quantity; and in some, blood. A writer commenting on these cases, says: "Now, these players, it is presumed, were specimens of perfect health, and violent muscular exercise was undoubtedly the great factor in producing the albuminuria, although mental excitement, cold bathing and nitrogenous diet exercised no inconsiderable influence as accessory causes. The albumin disappeared after a few hours. From the presence of casts, and even blood, there can be no doubt that an acute congestion of the kidneys was present in these cases, to which was added a weakened heart's action from fatigue, a concentrated urine from copious perspiration, and the irritating effects of an abundance of oxalates and uric acid, which conditions are the very ones which experimentation has shown to favor the production of albuminuria." He concludes by saying: "Such then are the consequences attending upon severe muscular exertion."

The class of cases due to some fault of nerve innervation, or neurotic, as they are called, seems a large one. Runeberg has attributed these cases to some disturbance of the action of the heart or blood-vessel through the influence of the vasomotor nerves, which may be either a dilatation from paralysis, or contraction of the arteries by cramp or spasm. He found that irritation of the renal nerves produced contraction of the small arteries and capillaries of the kidneys, and that albumin then appeared in the urine. The contraction of the small vessels retards circulation, decreases arterial blood, and causes anoxemia, or deficiency of oxygen in the blood. Other experiments of his on the renal nerves caused vasomotor paralysis and dilatation of arteries, and slowed the circulation in the kidneys with a decrease or arrest of urinary secretion, and appearance of albumin in the urine. Violent irritation of the skin, such as burns, severe applications or severe chilling cause albuminuria. Irritation of the auditory nerve by rapid detonations; of the retina by strong light; of the solar plexus by hypodermic injections of chloroform, all cause a transient albuminuria. Anger, simple nervous excitement, prolonged mental action, and many other slight causes result in the same. It seems quite probable then that many of the cases of transient albuminuria we meet, and are in doubt as to the cause, may be due to some disturbance of the nervous system which controls the vasomotor nerves; and from what has been said, we have seen that vascular changes in the kidneys do result from this vasomotor disturbance.

The last of these albuminurias is the uric acid or oxaluric form. This form includes all those cases sometimes described as due to local or the mechanical cause, as Charcot calls it, irritation of the kidneys.

They are brought about, it is thought, by defective metabolism, assimilation and tissue waste. When we have a concentrated condition of the blood, as after violent exercise, or from products of defective assimilation, or bile, or specially from uric acid or oxalate of lime, we meet with these cases. They are generally somewhat nervous or dyspeptic, and this is said to be most apt to occur in the oxalate of lime cases. The specific gravity of the urine is apt to be high; may persist at 1.036, and generally deposits urates, uric acid and oxalate of lime. The amount of albumin is apt to be small and intermittent. Dr. Da Costa includes among these cases the albuminurias of adolescence, and says they present the same general features of the cases just described—*i. e.*, nervous and digestive disturbances, intermittent albuminuria, urine of high specific gravity, and deposits of urates and oxalates. He then asks whether this tissue waste, with its uric acid and oxalates may not cause functional albuminurias, and also whether the albuminuria of forced and excessive exercise may not be due to the same causes. The football players mentioned before all had uric acid, urate and oxalates, and some had casts covered with crystals. Dr. Da Costa believed the pathology of all these cases to be a congestion of the kidney, and if this continued he thought that local inflammatory changes occurred, due to the irritating effects of excreting the waste products. I think, however, it is now thought that most of the cases of functional albuminuria are due to a combination of the various causes mentioned. But whatever may be the cause or causes, they all seem to produce a local vascular change in the kidney.

It may be asked, "How can this be definitely proved, since true functional albuminuria is rarely fatal, and we cannot make observation after death?" It is true, we cannot make experiments on man, but it can be done on animals; and these experiments all indicate that vascular changes do take place in the kidneys. Then Millard and Shattuck, after many studies of kidneys after death, reported that it was rare not to find some scar or contraction or destraction of Malpighian bodies (or glomerulites), or other evidence of kidney trouble which had healed after local changes or disturbances in the kidney due to latent albuminuria.

Now in the light of our present knowledge, what are we to say about functional albuminuria?

Let me review rapidly: 1. There is a transient or functional albuminuria, occurring with greater or less frequency, and with more or less persistence. 2. These cases have been traced to various causes already enumerated. 3. Under whatever form these functional albuminurias occur, we have good reason for believing that vascular changes in the kidneys are always found. 4. It is believed that in most, if not all of these cases, there is a true local congestion of the kidneys, which may lead on to more serious trouble if persistent. 5. We have no reason for believing that there is a true physiological albuminuria—*i. e.*, that albumin is a normal ingredient of the urine, and is found, as Senator claims, in all healthy urines at all times.

If these conclusions are correct, they are most interesting to all medical men. It is believed then that in every case of functional albuminuria there is a real congestion of the kidney; slight it may be, and transient in some cases, but most acute in others, and if persistent, capable of leading on to further trouble. With our present knowledge, we are not able to predict which form may prove transient and harmless, or which lead on to chronic lesions. And until we can do this, it seems to me we are right in regarding these cases with suspicion. The general practitioner, I think, should keep these cases under observation, if possible, or if an examiner for a life insurance company, he should either decline the risks, or postpone with the hope of being able to make further study of the cases, or, as I believe is now done in some companies, consider albuminuria as involving an extra hazard, and then make use of endowment forms, short periods, or other forms of insurance to write insurance on these lives.

Some years ago I heard Dr. Paul Monde read a paper before the New York Academy of Medicine. Dr. Gaillard Thomas was on the platform near the president. Dr. Monde had scarcely taken his seat, after reading his paper, when Dr. Thomas jumped up, and said: "Mr. President and Gentlemen, there is nothing new in the paper of the evening."

Now I am quite willing to acknowledge that nothing new or original has been presented to-night, and yet I thought it well to try to review some things which have been written on this subject during the past few years; and in the discussion which I hope may follow, I feel quite sure I will learn something.

